# Supplementary material for: Locking loop movement in the ubiquinone pocket of complex I disengages the proton pumps
Source: Nat Commun. 2018 Oct 29;9:4500. doi: 10.1038/s41467-018-06955-y (PMC6206036; doi:10.1038/s41467-018-06955-y)
Supplement: Supplementary file 1 — Supplementary Information [file 41467_2018_6955_MOESM1_ESM.pdf]

Supplementary Information

Locking loop movement in the ubiquinone pocket of complex I disengages the proton pumps

Alfredo Cabrera-Orefice, Etienne Galemou Yoga, Christophe Wirth, Karin Siegmund, Klaus Zwicker, Sergio Guerrero-Castillo, Volker Zickermann, Carola Hunte and Ulrich Brandt

a

b

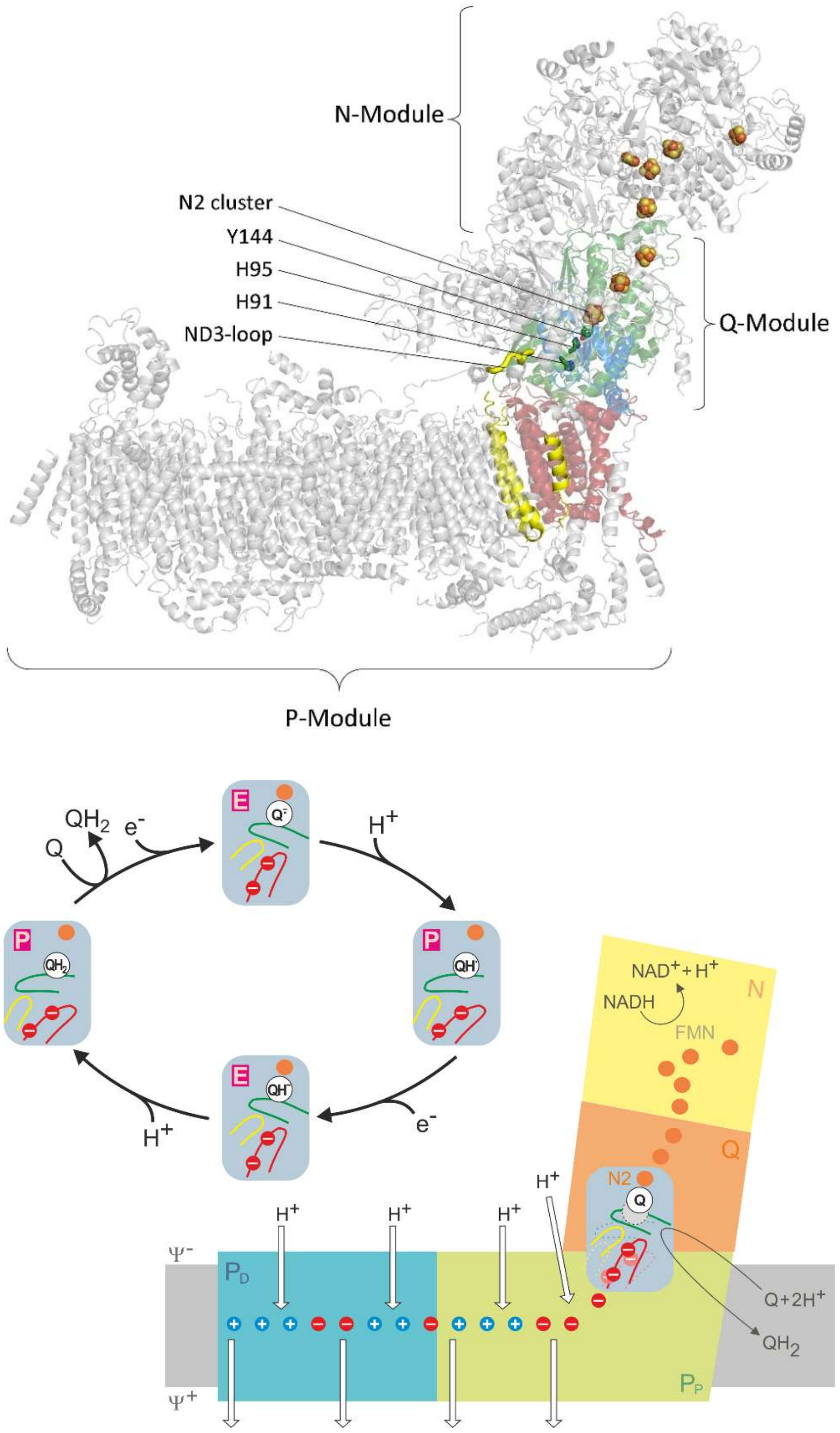

Supplementary Fig. 1: *Overall structure, functional modules and mechanistic model of complex I.* **a.** X-ray structure of complex I from *Yarrowia lipolytica* mutant Q133C^{PSST} showing its modular organization. ND1 (red); ND3 (yellow); 49-KDa (green) and PSST (blue). Residues Y144, H91 and H95 of the 49-kDa subunit involved in ubiquinone binding are shown in stick representation; iron-sulfur clusters, including the immediate electron donor for ubiquinone cluster N2, are shown in space fill representation. **b.** Cartoon of the functional modules and catalytic cycle of the hypothetical stabilization change mechanism[1,2]. In the ubiquinone pocket (grey box), the mobile loops β_1-$\beta_2^{49k\text{-}Da}$ (green), TMH1-2^{ND3} (yellow) and TMH5-6^{ND1} (red) are shown. Q, $Q^{\bullet -}$, $QH^{\bullet}$, QH^-, QH_2, reaction intermediates of ubiquinone; E, E-state; P, P-state; orange dots, irons-sulfur clusters; + (blue), - (red), chain of protonable residues.

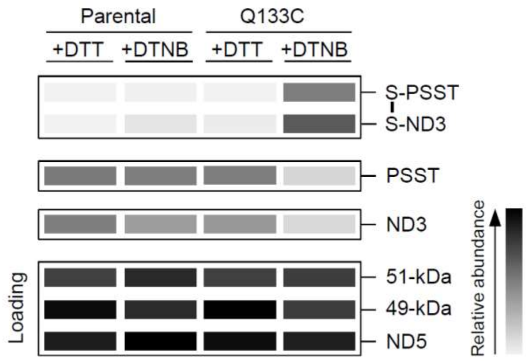

Supplementary Fig. 2: *DTNB treatment almost completely cross-links subunits PSST and ND3 in complex I from mutant Q133C^{PSST}.* Purified complex I was treated with 5 mM DTT or 0.1 mM DTNB for 5 min and then separated by 16% Tricine-SDS-PAGE. Gel slices at around of the regions of interest were cut and analyzed by mass spectrometry. Relative abundances of subunits PSST and ND3 were normalized to the sum of IBAQ-values from subunits 51-kDa, 49-kDa and ND5, which were used as loading controls. Data are shown as gray-shaded bars with light gray representing minimal and black maximal averaged IBAQ-values as indicated in the scale bar on the right (n=3 technical replicates).

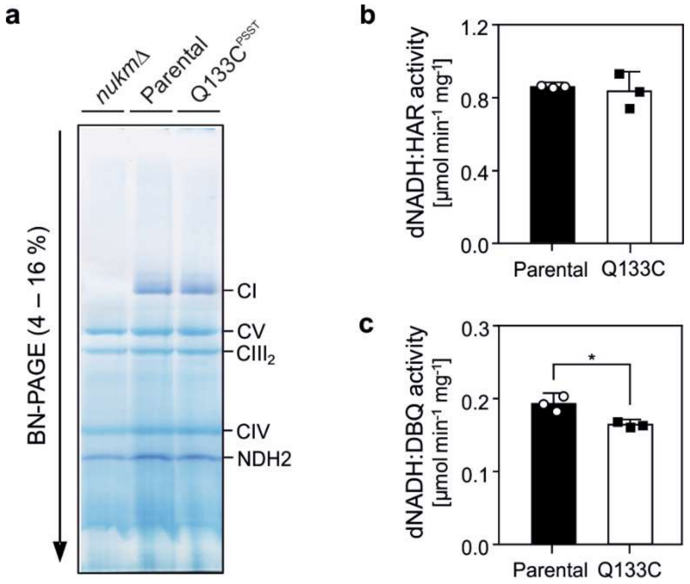

Supplementary Fig. 3: *Complex I assembly and activity are not affected by mutation Q133C^{PSST}.* **a.** DDM-solubilized mitochondrial proteins from strains *nukm*Δ, parental and Q133C^{PSST} were separated by BN-PAGE. In-gel NADH dehydrogenase activity staining revealed the positions of complex I (CI) and alternative NADH-dehydrogenase (NDH2). Other Coomassie-stained complexes of oxidative phosphorylation were identified by their characteristic migration pattern: complex III ($CIII_2$), complex IV (CIV), complex V (CV); **b.** dNADH:HAR oxidoreductase activities in untreated mitochondrial membranes indicated unchanged complex I content and a fully functional NADH oxidation site (mean ± s.d.; n=3 independent experiments). **c.** A ~15% decrease in DQA-sensitive dNADH:DBQ oxidoreductase activity was observed in untreated mitochondrial membranes from mutant Q133C^{PSST}. Residual unspecific dNADH:DBQ activity (0.009 µmol min^{-1} mg^{-1}) measured in strain *nukm*Δ containing no complex I was subtracted from the rates obtained with membranes from parental and mutant strain (mean ± s.d.; n=3 independent measurements; *p<0.05, ANOVA with Bonferroni correction).

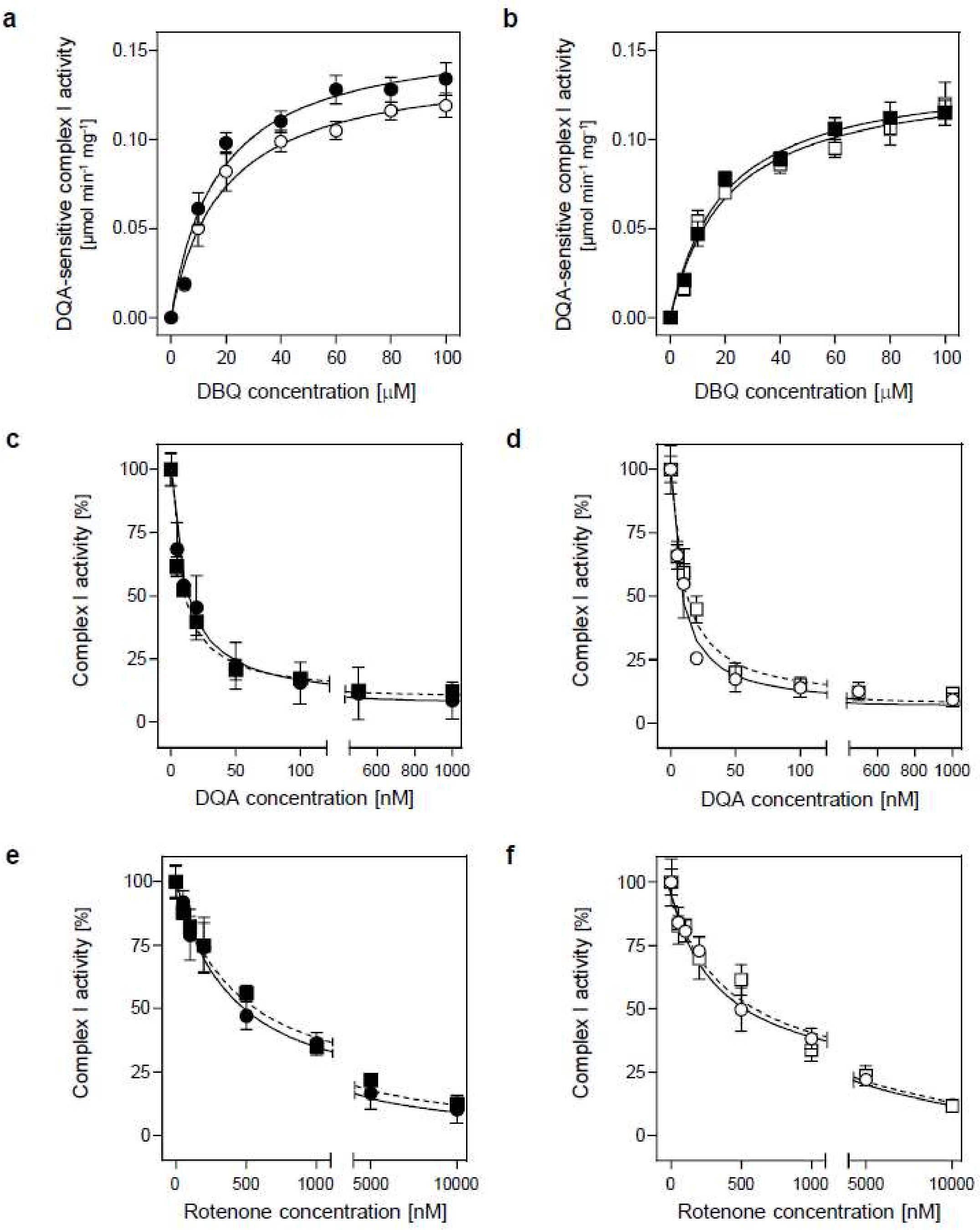

Supplementary Fig. 4: *Michaelis-Menten kinetics and inhibitor sensitivity of complex I were unaffected in mitochondrial membranes from mutant Q133C^{PSST}.* **a., b.** Michaelis-Menten plots for the substrate DBQ. Inhibitor-sensitive dNADH:DBQ oxidoreductase activities were measured with mitochondrial membranes from parental strain **(*a*)** and Q133C^{PSST} **(*b*)** treated with 5 mM DTT (filled symbols) or 0.1 mM DTNB (open symbols) for 5 min before starting the reaction. **c.-e.** Inhibition of complex I oxidoreductase activity by increasing concentrations of DQA **(*c, d*)** and rotenone **(*e, f*)** in membranes from parental (circles, solid lines) and mutant strain (squares-dashed lines) treated with DTT (filled symbols) or DTNB (open symbols). In panels ***c-f***, complex I activities were normalized to their respective contents as assessed by their dNADH:HAR oxidoreductase activities and values in the absence of inhibitors were set to 100%. In all experiments, dNADH concentration was fixed to 100 µM. Complex I activities in the presence of inhibitors were measured with a DBQ concentration of 100 µM. Data from three independent batches of mitochondrial membranes (mean ± s.d.) are shown.

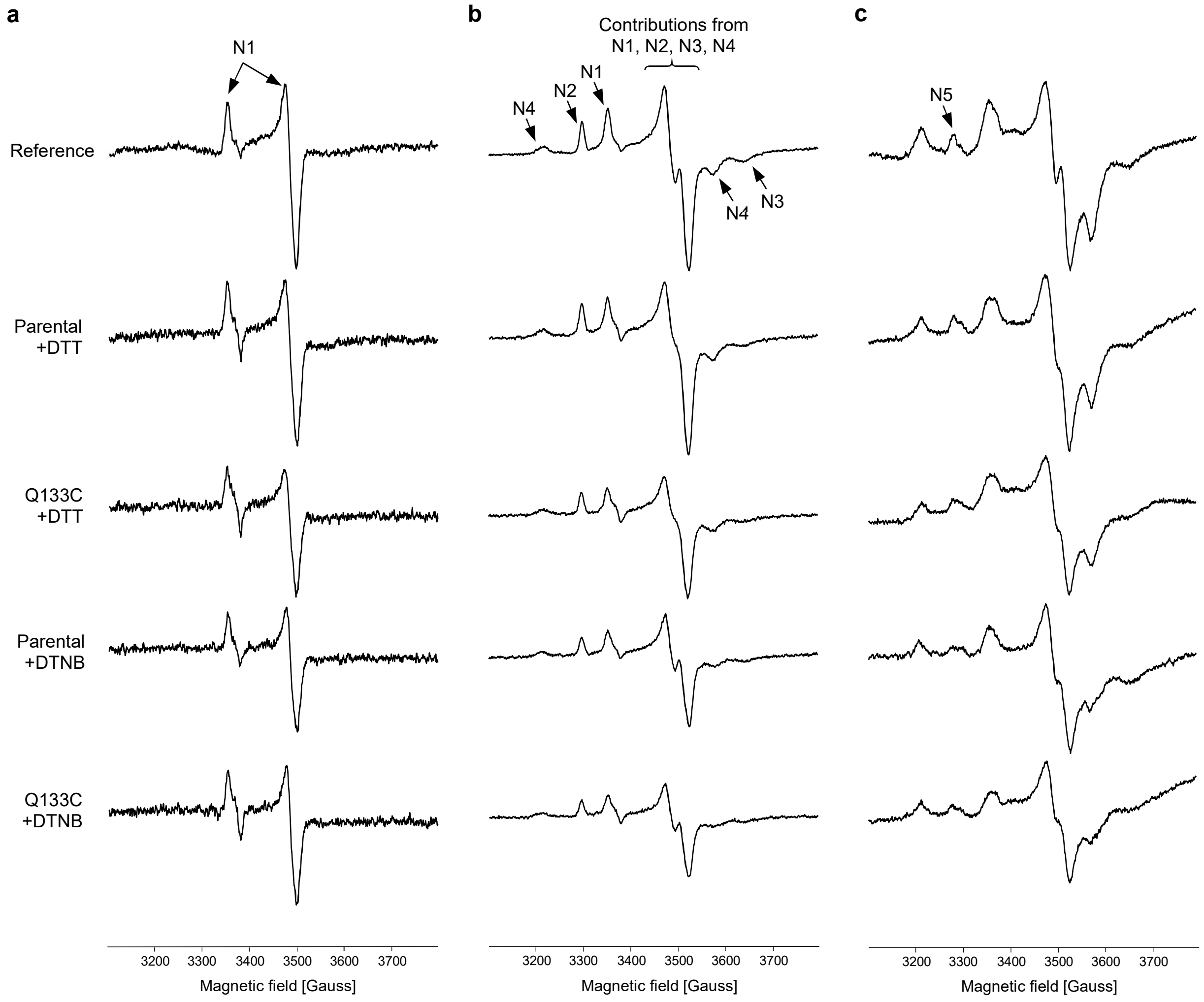

Supplementary Fig. 5: *EPR spectra of purified complex I from parental strain and mutant Q133C^{PSST}.* cw-EPR spectra recorded from NADH reduced complex I samples treated with either DTT or DTNB at temperatures 40 K (***a***), 12 K (***b***) and 5 K (***c***). EPR conditions: microwave frequency 9.475 GHz, modulation amplitude 6.4 G, microwave power 1 mW (40 K and 12 K) or 20 mW (5 K). Characteristic signal contributions of individual iron sulfur clusters are indicated by arrows.

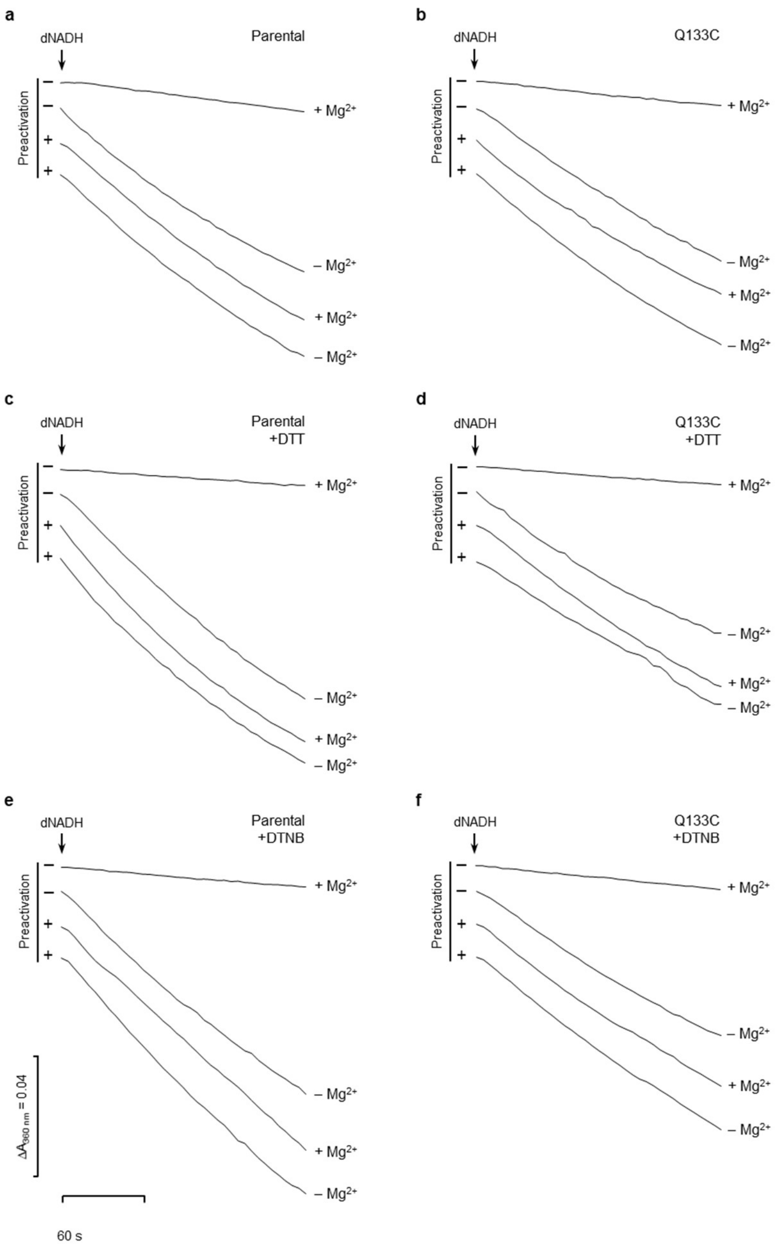

Supplementary Fig. 6. *Reactivation of complex I in mitochondrial membranes from parental and Q133C^{PSST} strains is blocked in the presence of Mg^{2+}.* dNADH:DBQ oxidoreductase activities were measured in the absence or presence of 5 mM $MgCl_2$. In all cases, 1 mM DBQ was added to the samples (see methods) before the reaction was started by the addition of DBQ activity buffer containing 110 µM dNADH with or without 5.5 mM $MgCl_2$. dNADH oxidation was monitored for 3 min at 25°C. In order to fully activate complex I in membranes, samples (25 µl) were preactivated with a small pulse of 5 µM dNADH in the presence of 1 mM DBQ and 1 mM NaCN within 1 min before adding DBQ activity buffer supplemented with $MgCl_2$ and starting the assay. The final concentration of DBQ and membranes were 100 µM and 50 µg ml^{-1}, respectively. dNADH oxidation traces obtained from parental (***a***) and mutant Q133C^{PSST} (***b***) samples "as prepared". Mitochondrial membranes were treated with 5 mM DTT (***c, d***) or 0.1 mM DTNB (***e, f***) for 5 min before preactivation and starting the assay. Complex I activities indicated in Fig. 2 (non-preactivated) correspond to the linear initial dNADH oxidation rates, which were calculated by taking the slopes between 5-30 s or 60-90 s in the absence or presence of Mg^{2+}, respectively. dNADH oxidation traces from samples with (+) or without (-) preactivation step are indicated. Representative traces of experiments performed with one out of three independent batches of mitochondrial membranes are shown.

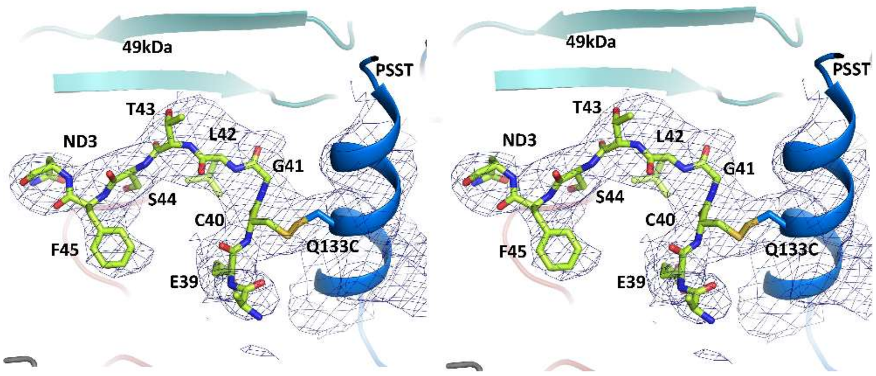

Q133C

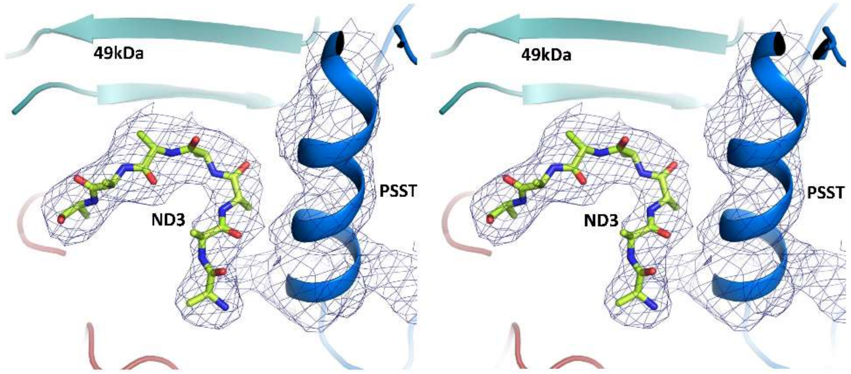

Wild Type

Supplementary Fig. 7: *The tip of the ND3 loop connecting TMH1 and TMH2 adopts a same conformation in wild type (PDB 4W27) and mutant Q133C^{PSST} complex I structures.* (**top**) Close-up view of the structure of mutant Q133C^{PSST} complex I at the position of the disulfide bond between C40^{ND3} and Q133C^{PSST} (stereo view). The 2Fo-Fc electron density map (blue mesh) is contoured at 1.0σ. The color code is as in Fig. 4. (**bottom**) Similar stereo view of wild type complex with the corresponding 2Fo-Fc electron density map contoured at 1.0σ showing the ND3 loop connecting TMH1 and TMH2. Note that for the wild type, the electron density was less well defined in this area not allowing side chain assignments, although this structure had a slightly higher resolution than the structure of the Q133C^{PSST} complex I

Supplementary Table 1. *Data collection and refinement statistics.*

	Q133C^{PSST}	Q133C^{PSST} (5.975 keV)	Wild type (5.200 keV)
**Data collection**			
Space group	H32	H32	H32
Cell dimensions			
a, *b*, *c* (Å)	316.3, 316.3, 819.2	312.9, 312.9, 817.0	318.2, 318.2, 824.1
α, β, γ (°)	90.0, 90.0, 120.0	90.0, 90.0, 120.0	90.0, 90.0, 120.0
Resolution (Å)	50-3.79 (3.99-3.79)*	50-5.0 (5.1-5.0)	50-5.1 (5.1-5.0)
R_{merge} (%)	20.4 (530.7)	19.1 (236.2)	15.6 (186.7)
CC(1/2) (%)	100.0 (43.9)	99.6 (72.6)	99.9 (91.3)
$I / \sigma I$	13.2 (0.7)	10.09 (1.84)	23.93 (3.64)
Completeness (%)	99.6 (97.7)	99.9 (100.0)	99.9 (99.9)
Multiplicity	21.0 (21.4)	57.5 (43.1)	53.5 (54.3)
**Refinement**			
Low resolution (Å)	40		
Anisotropic high resolution (Å)	3.79 x 4.2 x 4.2		
No. reflections	120,019		
R_{work} / R_{free}	36.1/36.3		
No. atoms			
Protein	35570		
Ligands	56		
B-factors			
Protein	165.3		
Ligands	202.1		
R.m.s. deviations			
Bond lengths (Å)	0.009		
Bond angles (°)	1.23		

Datasets were collected from one crystal each.
*Values in parentheses are for highest-resolution shell.

Supplementary Table 2. *List of primers used in this study.*

Primer name	Sequence (5'→3')
ST1_P1	CGTGTCTCCATCTGTCTCGAATGACCTTTG
ST1_P2	CATTACCCTGTTATCCCTAGCAATTGACCAATATCAAGGTGTAAAGATGACGATGATG
ST1_T1	CTAGGGATAACAGGGTAAGGCCAACCGGGCTTAGATAGAATCATAC
ST1_T2	CTTGTATTCTAATCGTGCCTAGCAAGGAAGTC
NUKM_Q133Cf	TGTGTGTACGACCAGATGCCCGAG
NUKM_Q133r	ACGCAGCACGGGGGCC
NUKM-seq-1fw	CTGTCACCAGCTTGTCTCG
NUKM-seq-2fw	CCCTGACCACTCTGGATGC
nukmleu2_fw2	GTCTCGTTCTCTTCACACCAGGCC
nukmleu2_rv2	GTACAGTCACTTGTACTCGTACCG
nukm5UTR_fw	CTCCTCTCATGGTCATTCCC
leu2inv_rv	GACGCATTGATGGAAGGAGC

References

1. Zickermann, V. et al. Mechanistic insight from the crystal structure of mitochondrial complex I. *Science* **347**, 44-9 (2015).
2. Brandt, U. A two-state stabilization-change mechanism for proton-pumping complex I. *Biochim Biophys Acta* **1807**, 1364-9 (2011).
